# Femoral exostosis causing vastus medialis pain in an active young lady: a case report

**DOI:** 10.1186/s13104-015-1077-0

**Published:** 2015-04-02

**Authors:** Neil Heron

**Affiliations:** Department of General Practice and Primary Care, Queen’s University Belfast, Belfast, Northern Ireland; Centre for Public Health, Queen’s University Belfast, Belfast, Northern Ireland; UKCRC Centre of Excellence for Public Health (NI), Queen’s University Belfast, Belfast, Northern Ireland

**Keywords:** Femoral exostosis/osteochondroma, Hereditary multiple exostoses, Medial thigh pain, Malignant transformation

## Abstract

**Background:**

Musculoskeletal conditions are a common reason for consultation to General Practitioners (GPs)/family physicians in primary care. Osteochondromas are the most common benign bone tumours and usually occur in the metaphyseal region of long bones. Despite the distal femur being the commonest location to find these benign bone tumours, this is the first case report in the literature specifically describing vastus medialis muscle pain as the presenting symptom due to underlying bursa formation secondary to local pressure effects.

**Case presentation:**

Twenty nine year old female of white British ethnic origin, presenting to a primary care clinic with a three year history of intermittent left distal medial thigh pain.

**Conclusion:**

The benign bone tumour, femoral exostosis/osteochondroma, was diagnosed via Magnaetic Resonance Imaging (MRI) and treated conservatively, with surgical excision an option if not resolving. GPs/family physicians need to be aware of this diagnosis and that femoral exostosis/osteochondroma can present to primary care physicians, particularly within the second decade of life.

## Background/Discussion

An osteochondroma or exostosis is a benign bone tumour consisting of a bony overgrowth that occurs commonly in the metaphysis of long bones and pelvis [1], although any bone can be affected [2]. They are the most common of the benign bone tumours [3], occurring in approximately one per cent of the population [4-7]. Osteochondromas can be solitary, as in approximately seventy-five per cent of cases [8], or mutlple, presenting as part of the autosomal dominant condition, hereditary multiple exostoses [2,9-12], which is not a focus of this case report. Despite the fact that osetochondromas are most commonly found in the distal femur [13], this is the first report identifying symptoms within the vastus medialis muscle as the presenting features of this condition.

Osteochondromas can be discovered incidentally although complications are reported to occur in four per cent [12]. Musculoskeletal manifestations of osetochondromas have included hip impingement [14], limb deformities, fractures and localised pain or swelling of the affected area, including around the ankle [7]. Yoong *et al.* [14] also report a case–control study of exostoses causing ischiofemoral impingement. As in this case report, osteochondromas can cause local bursa formation [4,8], most frequently developing around areas which are mobile, which can cause local pain and swelling. Differentiating bursa formation from malignant transformation then becomes important and is discussed later in this case report. With approximately twenty per cent of a General Practitioners (GPs)/family physicians workload being related to musculoskeletal conditions [15], GPs/family physicians need to be aware that osteocohondromas are common and can present with musculoskeletal complaints, amongst others.

Indeed osteochondromas have also been previously documented to cause vascular injuries and symptoms [5], including popliteal artery compression [1] and rupture [12] as well as acute lower limb ischaemia [1,6]. Previous authors [16] have also presented twenty cases of osteochondromas causing peripheral nerve injuries, including sciatic nerve impingement from a distal femoral exostosis [3,11]. The commonest age of presentation is in the second decade of life [1,5], which coincides with skeletal maturity and ossification of the osteochondroma.

Some authors have used Computed Tomography (CT) to diagnose the condition [8,11] whilst others have used plain x-ray [1,8] and Magnetic Resonance Imaging (MRI) [1,2]. Duplex ultrasongraphy and angiograms may also be worth considering to exclude vascular injury [1,5,8].

With regards treatment options, the majority will settle with conservative and supportive measures [4]. If symptoms are unresponsive to conservative treatment, then surgical removal of the osteochondroma remains an option [5]. Some authors have undertaken surgical removal using an endoscopic approach, particularly within the distal femoral region [17]. Fitzgerald *et al.* [18] present a case report of how a patient with multiple osteochondromata got symptomatic relief from hip impingement secondary to hip joint exostosis, with surgical removal of the offending osteochondromas. Some multiple osteochondromas have also been symptomatic to the extent that they have required total hip and/or knee arthroplasty although the authors remind the surgeons that the anatomy is often distorted and therefore the surgery is more technically demanding [10]. Surgical options are generally postponed to after skeletal maturity [1,8] and patients need to be counselled pre-operatively regarding the two per cent recurrence rate following surgical removal of the lesion [4].

If patients and clinicians opt for conservative management, then they need to be aware of the potential for malignant change within osteochondromas, which is reported to occur in less than one per cent of such lesions [4,7,12], with malignant transformation being more common in hereditary multiple exostoses [10,12]. Malignant transformation can be signified by an increase in pain and swelling around the site as well as an increase in size of the mass [7,8]. MRI has been proposed as an unequivocal investigation in differentiating between benign and malignant changes [2,19] and thus can provide reassurance to the benign nature of the mass, as in this case report.

## Case presentation

A twenty nine year old white British female, who was a keen runner and currently in training for a half-marathon, presented to a General Practice (GP)/family physician clinic in primary care reporting a two to three year history of left medial thigh and knee pain. There was no obvious precipitating injury or trauma. The patient had initially presented to the minor injury unit within a local hospital and was then referred back to primary care with no specific diagnosis made. The left medial distal thigh pain would intermittently flare over the previous three years and the pain had flared over the preceding three days after simply bending her knees up in bed. There was no obvious specific precipitant to the pain and it had been occurring intermittently over this three year period. When it occurred, the pain was described as sharp and located over her left medial distal thigh, around the distal vastus medialis muscle region. The pain would last a few minutes and then settle with rest. There was no swelling, locking or giving way of the knee.

There was no past medical or family history of note and she was taking no regular medication. She was a non-smoker and drank less than ten units of alcohol per week. She had increased her running mileage over the previous three weeks from approximately ten miles to fifteen miles per week in preparation for the half-marathon but this had no influence on the pain she was experiencing. The pain was not stopping her from training approximately six hours per week.

### Examination

On initial inspection with the patient wearing shorts, there was obvious bilateral flat feet and evidence of overpronation on walking. Walking was pain-free with no obvious limp. Walking on heels and tip-toes was normal. Lunging, squats and one-legged squats were normal and pain-free. With the patient standing and then lying down, there was no other obvious limb malalignment, joint swelling or loss of muscle bulk in the different leg compartments. All hip and ankle range of movements, both active and passive, were pain-free and full. During palpation, there was tenderness and crepitus palpated within the head of the vastus medialis muscle. No other abnormality was palpated and no bony tenderness was palpated. Patella tap of both knees was negative.

Both active and passive movements of both knees were full and pain-free. All resisted movements of the lower limb were of full strength and pain-free. All ligament tests within the knees, including collateral and cruciate ligaments, were pain-free and within normal limits. There was no meniscal pathology identified on McMurray’s or Apley’s tests. Patellar apprehension testing was normal as was Ober’s test for iliotibial band friction syndrome.

### Differential diagnosis

Following the history and examination, the working diagnosis was a muscular strain of the left vastus medialis muscle although the history and examination did not entirely fit with this diagnosis. Other diagnoses to consider with medial knee pain include medial collateral ligament sprain, medial meniscus pathology, pes anserine bursitis, medial facet patella pathology and medial plica syndrome.

After discussion with the patient, it was therefore agreed to undertake an MRI of the left thigh and knee region to allow further investigation of this presentation. A plain x-ray was not performed at this stage as no bony tenderness or trauma was elicited. A MRI of the left knee and thigh region was performed.

### Diagnosis

**Figure 1:**
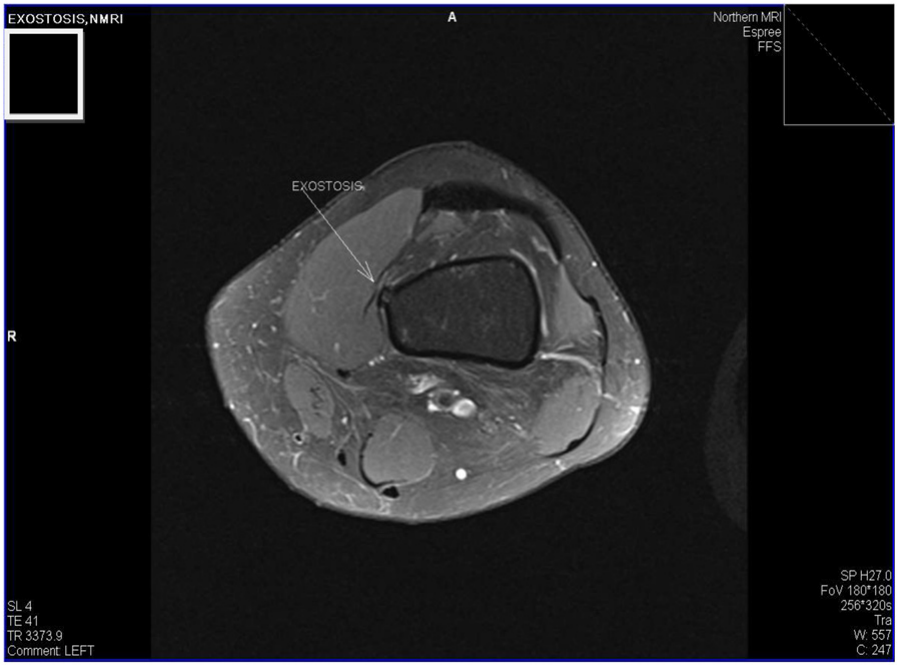
**Arrow illustrating the benign exostosis within the distal femur.**

**Figure 2:**
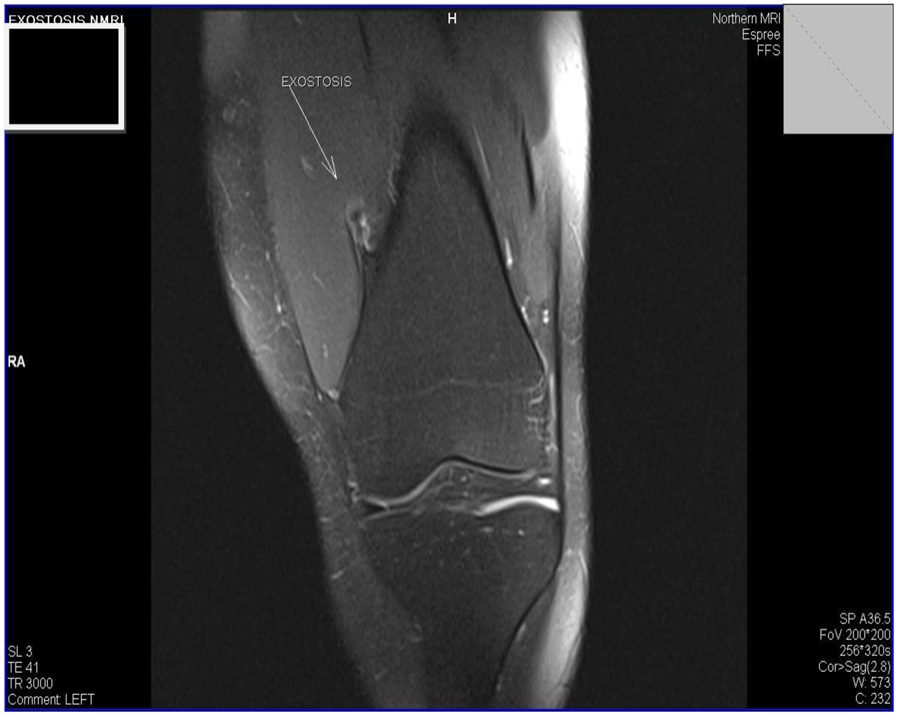
**Arrow illustrating the benign exostosis within the distal femur and surrounding bursa.**

The MRI (Figures 1 and 2) was reported as a small benign exostosis protruding into the left vastus medialis muscle causing a small pseudo-bursa and oedema within the muscle as a result.

### Treatment

The management of the condition was discussed with the patient, including with an orthopaedic surgeon, and she opted for conservative management, with surgical options to be considered if her symptoms were not resolving.

## Conclusion

Osteochondromas are an infrequent but important cause of musculoskeletal presentations, particularly in patients presenting with such symptoms around the second decade of life and may present to General Practitioners (GPs)/family physicians in primary care, who commonly deal with musculoskeletal symptoms. The investigation of choice for musculoskeletal symptoms appears to be MRI preceded by simple x-ray, with MRI helping to exclude malignant change within the bony mass if this is suspected. Treatment options include conservative options and surgical removal when this fails. Musculoskeletal doctors, including GPs, need to be aware of this potential diagnosis in patients presenting with uncommon musculoskeletal symptoms.

## Consent

Written informed consent was obtained from the patient for publication of this Case Report and any accompanying images. A copy of the written consent is available for review by the Editor-in-Chief of this journal.

## Abbreviations

CT: Computed tomography
GP: General practitioner (s)
MRI: Magnetic resonance imaging
